# Brain neural patterns and the memory function of sleep

**DOI:** 10.1126/science.abi8370

**Published:** 2021-10-28

**Authors:** Gabrielle Girardeau, Vítor Lopes-dos-Santos

**Affiliations:** 1 Institut du Fer a Moulin, UMR-S 1270 INSERM and Sorbonne Université, 75005 Paris, France; 2 Medical Research Council Brain Network Dynamics Unit, Nuffield Department of Clinical Neurosciences, University of Oxford, Oxford, OX1 3TH, UK

## Abstract

Sleep is crucial for healthy cognition, including memory. The two main phases of sleep, REM and Non-REM sleep, are associated with characteristic electrophysiological patterns recorded using surface and intracranial electrodes. These patterns include sharp wave-ripples, cortical slow oscillations, delta waves and spindles during Non-REM sleep, and theta oscillations during REM sleep. They reflect the precisely timed activity of underlying neural circuits. Here, we review how these electrical signatures have been guiding our understanding of the circuits and processes sustaining memory consolidation during sleep, focusing on hippocampal theta oscillations and sharp wave-ripples and how they coordinate with cortical patterns. Finally, we highlight how these brain patterns could also sustain sleep-dependent homeostatic processes and evoke several potential future directions for research on the memory function of sleep.

Memory formation is the challenging process of selecting which new experiences will be stored and integrated into an existing structure of memories that needs to be simultaneously preserved and modified. During wakefulness, this occurs concurrently with an uninterrupted flow of new sensory experiences. Sleep provides a window of opportunity for the brain to sort and reinforce newly encoded memories in absence of the incessant barrage of external information. This process, called “consolidation”, leads to the generation of long-lasting “memory traces” or “engrams” whose activation during wakefulness supports the recall of information.

During sleep, a myriad of neural networks involved in memory processing are endogenously activated. Their activity generates electrical potentials captured using noninvasive surface electrodes (EEGs) or intracranial electrodes which can record local field potentials (LFPs) as well as action potentials (spiking activity). A large amount of effort has been devoted to describing how we can use meaningful patterns in these electrical fluctuations to understand the brain. These patterns include oscillations (eg. theta rhythm), transient potentials with an identifiable waveform (eg. dentate spikes), and spiking activity patterns (eg. UP and DOWN states). Combining signal analysis, anatomical data as well as targeted intracranial recordings or manipulation of superficial and deep structures has boosted our understanding of the cellular basis of these patterns. Ultimately, these advances may lead to an understanding of the role that sleep brain patterns play in learning and memory.

## NREM sleep and hippocampal sharp-wave ripples

**Fig. 1:**
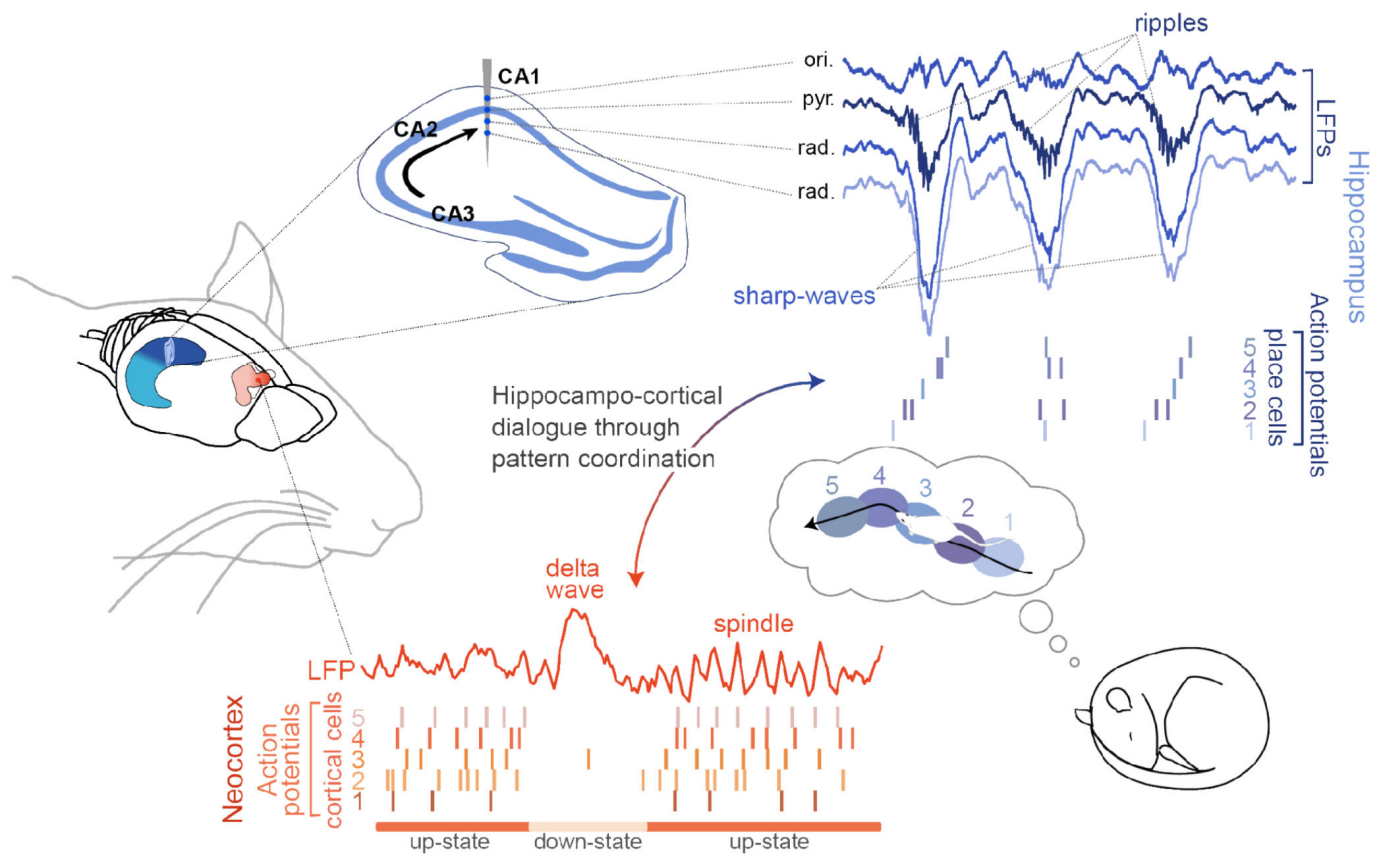
Hippocampal and cortical patterns coordinate during NREM sleep to sustain memory consolidation. In the hippocampus, coordinated input from CA3 depolarizes CA1 pyramidal neurons to create a sharp-wave in the radiatum layer (rad.) and a fast, 200Hz ripple in the pyramidal layer (pyr.). SWRs are associated with place cell activity that recapitulates the trajectories experienced in the previous wakefulness epoch. In the neocortex, unit activity alternates between periods of high activity (UP state) associated with spindles, and silence (DOWN state) reflected on the LFP as a delta wave.

**Fig. 2:**
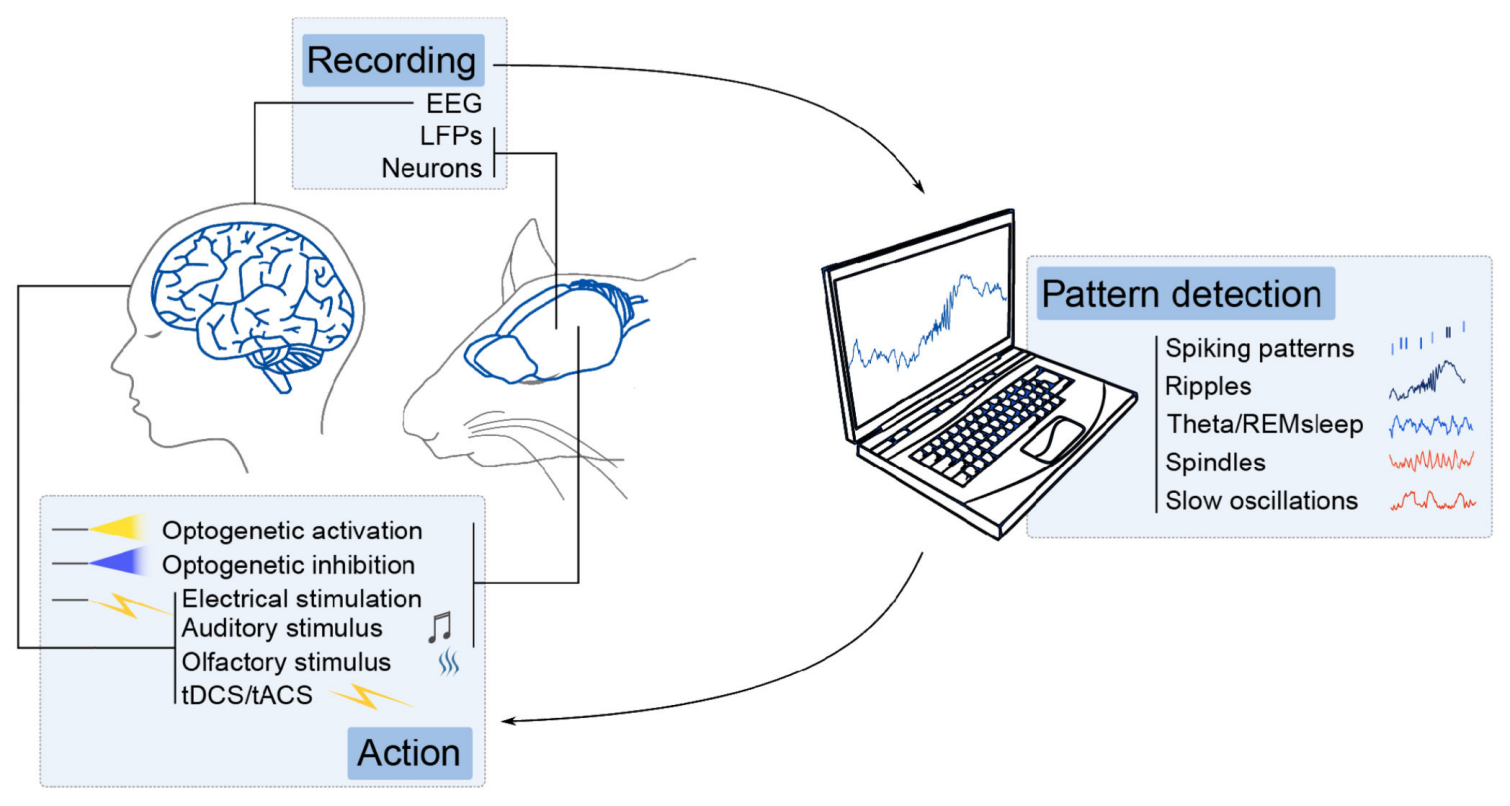
Closed-loop experiments allow for the modulation of ongoing brain patterns in real-time. Recorded brain signals are processed in real-time to detect sleep patterns. The detection of a given pattern automatically triggers an action using invasive or noninvasive methods that affect the neural networks in real time to test whether the manipulation boosts or impairs memory consolidation. The effect on memory is assessed during a recall session following the modified sleep period.

One of the most important patterns in sleep is the sharp-wave ripples (SWR) complex (Fig. 1). The hippocampus is a 3-layer structure in which the information flows from the dentate gyrus to the CA1 region through CA3. During sleep, CA3 pyramidal neurons spontaneously activate in synchronous bursts that trigger a massive activation of CA1 pyramidal cells. In the stratum radiatum the CA3 input on pyramidal cell dendrites creates the sharp wave, while in the CA1 pyramidal cell layer, the interplay between activated pyramidal cells and interneurons gives rise to the fast (100-250Hz) oscillatory part of the event: the ripple (1). The two-step theory (2) postulates that first, a subgroup of CA3 and CA1 cells are coordinated by theta oscillations during an experience and form cell assemblies encoding the corresponding new information. Then, in subsequent sleep periods, these CA3 assemblies spontaneously ignite SWR events reactivating the associated CA1 ensembles and promote the strengthening of their connections which ultimately leads to memory consolidation. Consistent with this theory, pairs of CA1 pyramidal cells that cofire during the exploration of an open field maintain this correlation during subsequent sleep SWRs (3). The persistence of the activity correlations observed in awakening in subsequent sleep is commonly referred to as sleep *reactivation*. Using a wide range of methods (4, 5), subsequent studies established that cofiring patterns and entire sequences of place cells activated during wakefulness are reinstated during the SWRs of the following sleep epoch (“*replay*”, (6) Fig. 2). Importantly, reactivation was also shown in humans (7).

**Fig. 3:**
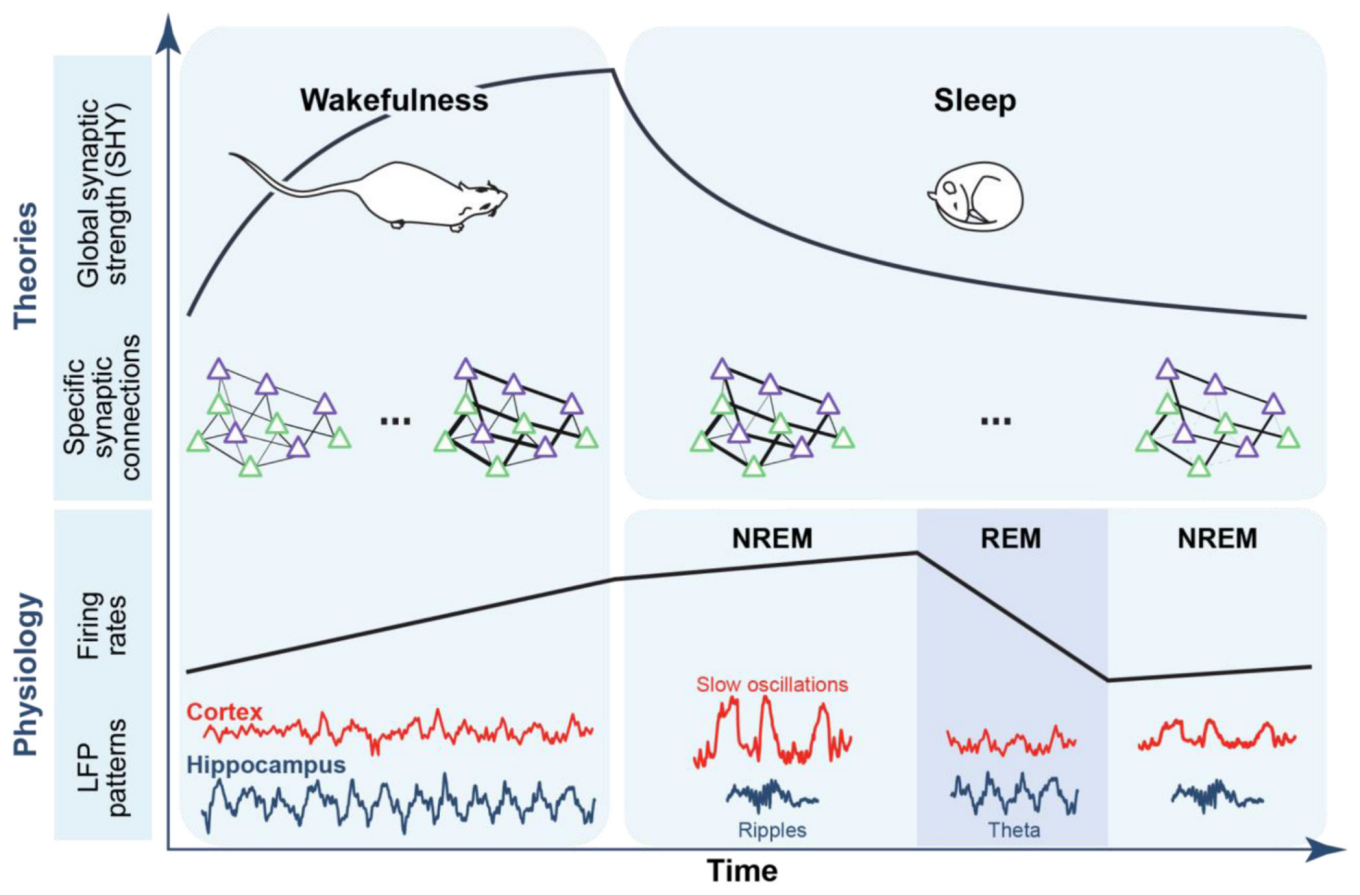
Homeostasis and memory consolidation may occur in parallel across wake-sleep cycles. During learning, synapses are globally enhanced and overall firing rates progressively increase as the brain encodes new information into cell assemblies (engrams: green and purple triangles; the thickness of the black line represents the strength of the connection) paced by theta oscillations in the hippocampus. During extended sleep periods including sequences of NREM and REM epochs, homeostatic processes involving cortical slow oscillations and REM sleep theta combine to downscale overall firing rates and global synaptic strength in accordance with SHY. In parallel, the specific connections among cell assemblies are selectively consolidated through ripple-related temporally organized reactivation (see fig. 1).

The first causal studies for the role of reactivation in memory consolidation developed closed-loop paradigms (Fig. 3) to disturb sleep ripples, and therefore the associated reactivation. They showed drastic spatial memory impairment (8, 9). Optogenetic silencing of CA1 pyramidal neurons during sleep SWRs following the exploration of novel environments impairs the reinstatement of these cell assemblies upon reexposure to the same environment, suggesting memory impairments at recall are due to a lack of consolidation of the spatial maps, or engrams, sustaining the memory (10). Various factors influence SWRs-associated reactivation during sleep. For example, reactivation in CA1 is stronger, and lasts longer (11) after novelty. It is also biased towards the activity previously expressed in theta cycles associated with strong mid-gamma oscillations (50-100 Hz), suggesting that assemblies formed during the heightened influence of entorhinal cortex, thought to convey new extra-hippocampal information, is preferentially reactivated (12). Most SWRs and reactivation studies focus on CA1. However, social memory traces are reactivated in CA2 during SWRs, and their bidirectional modulation enhances or impairs social memory (13). These results suggest that while CA3 might bias the SWR reactivated assemblies to consolidate spatial memories, CA2 is essential to bias SWR content towards social memories.

The development of algorithms for fast, online detection of specific replay content, as opposed to the mere detection of ripples on LFPs, is a necessary step to further our understanding of the role of sleep replay. Along this line, Gridchyn et al. (14) trained rats to forage in two environments and disrupted the following sleep and rest SWR events except the ones reactivating the first environment. The performance on this environment was better than on the second one, indicating that the consolidation of the spatial memories related to the first environment were spared from disruption. Altogether, the results accumulated over the last decades strongly indicate that reactivation of hippocampal ensembles associated with novel information and learning during sleep SWRs is essential for memory consolidation. Surprisingly however, it is still unknown whether hippocampal reactivation also occurs in the ventral part of the hippocampus, which has different connectivity and is involved in stress and anxiety. In addition, hippocampal dentate spikes reflecting strong cortical inputs to the dentate gyrus during NREM sleep have been identified as potential players in the NREM consolidation processes but remain to be further explored (15).

While this review focuses on sleep, SWRs also occur during awake immobility and non-exploratory behaviors (grooming, eating…). Although there are no clear qualitative differences between awake and sleep ripples, their replay content differs (6). A major challenge will be to understand if and how NREM sleep background (neuromodulation, reduced external inputs, cortical and subcortical NREM-specific activity, etc) makes sleep ripples and their associated neuronal content functionally different from the awake ones. Further, these differences could either be characterized as a simple sleep/wake dichotomy or occupy a multidimensional functional space (consolidation, forgetting, planning, memory reorganization, decision making, etc.) depending on numerous parameters including neuromodulatory levels, attention or alertness, ongoing behavior, sleep debt, circadian rhythm, consolidation needs, immediate and long-term previous experience, or NREM sleep substages.

## Hippocampo-cortical coordination through NREM sleep patterns

All major theories for long-term memory consolidation involve communication between the hippocampus and the neocortex (16). During NREM sleep, cortical circuits undergo an alternation of periods of marked high and low population activity, referred to as UP and DOWN states, respectively. This alternation translates in LFPs as the NREM sleep canonical slow oscillation. In particular, DOWN states are associated with distinctive LFP deflections called delta waves. Delta waves are often followed by spindles, bouts of 10-15Hz oscillations originating from the thalamus. All of these cortical rhythms have, individually, but mostly through their coordination with other hippocampal and cortical patterns, been related to memory consolidation (16–18) (Fig. 2).

Transcranial stimulation in humans can be used to boost slow oscillations during NREM sleep and the manipulation enhances performance at retrieval on the next day (19). Numerous EEG correlational studies have highlighted the importance of slow waves and spindles for memory consolidation (16). In rodents, interesting insights have emerged from a brain-machine interface experiment in which animals are trained to control a reward-delivering device by self-modulating the firing of a predefined set of neurons. Neurons causally involved in the task synchronized their firing around the UP phase of slow waves during subsequent sleep epochs. Further, the performance improvement at retrieval could be predicted by this synchrony increase and was impaired by specific optogenetic silencing of activity during the UP phase of the slow waves (20). Most cortical studies have focused on UP states while largely ignoring silent phases. Indeed, the way we study the brain suffers from technical, statistical, and conceptual biases and we tend to look at what we can most easily record and decode: periods of high population activity, higher firing neurons, salient oscillatory patterns. An original approach both using and getting around these biases showed that the very sparse, usually dismissed activity in the prefrontal cortex during the prominent delta waves (down states) actually reactivated cell assemblies formed during preceding learning (21).

SWRs and cortical NREM sleep patterns are temporally coordinated in a manner that is believed to promote plasticity and long-term consolidation of contextual (or episodic) memories (16, 22). Hippocampal SWRs incidence is increased at transitions to cortical UP and DOWN states and spindles troughs, and coordinated reactivations occur between the hippocampus and various cortical areas during SWRs (23). Indeed, enhancing hippocampo-cortical coordination by using a closed-loop system (Fig. 3) to generate a down-state/spindle complex after SWRs improves performance on a memory task (17). Optogenetically generating artificial spindles in coordination with hippocampal ripples and slow cortical oscillations also improves memory (18), highlighting the importance of the ripple/delta/spindle trifecta coordination for memory consolidation. Further, the spiking content of hippocampal SWRs can predict cortical firing in subsequent delta waves, suggesting that hippocampal SWRs bias the reactivated information in the cortex (21). On the other hand, cortical firing can also predict the reactivated content in CA1 (24), and sensory stimulation during sleep can bias the content of hippocampal reactivation and improve memory, a phenomenon called “targeted memory reactivation” (25). Altogether, these findings indicate that memory consolidation involves loops where cortical areas can bias memory traces reactivated in hippocampal SWRs, which in turn would evoke the reactivation of related multimodal representations in the neocortex.

## Beyond the hippocampo-cortical sleep-talking

Because of the robust conceptual framework provided by both the two-step consolidation theory and the idea of a gradual transfer of information from the hippocampus towards cortical areas, the majority of studies on sleep patterns and memory consolidation have focused on the hippocampo-cortical dialogue. However, many other structures are involved in memory formation. SWRs in the hippocampus are extremely powerful events that can synchronize activity across structures beyond the neocortex, potentially associating other features, such as emotional tone, to various forms of memories, during the consolidation process. For example, reward-located hippocampal place cells and reward-encoding ventral striatum neurons fire together during sleep SWRs following the rewarded experience, with hippocampal activity leading the striatal activity (26). Dorsal vs. ventral hippocampus SWRs modulate distinct populations of neurons in the nucleus accumbens (27), another crucial structure for reward processing. In the basolateral amygdala, a major center for valence encoding, a subset of neurons is modulated during hippocampal SWRs. The joint hippocampal-amygdala neuronal representation established during an aversive spatial experience is reinstated during the following NREM (but not REM) epoch, specifically during SWRs (28). These results suggest that hippocampal SWRs could be coordinators of brain-wide, plasticity-enabling activity or reactivation allowing for the formation of distributed engrams across cortical, but also non-cortical areas.

## REM sleep and theta oscillations

Despite longstanding general interest in REM sleep stemming from its association with vivid dreaming in humans, the functional physiology of REM sleep has been understudied compared to NREM sleep. REM sleep EEG/LFP activity closely resembles awake activity: it was originally called “paradoxical” sleep for this very reason. Indeed, the dominant rhythm during REM sleep is the theta oscillation, characteristic 5-12 Hz waves most prominent in the hippocampus, but recorded in the cortex and other subcortical structures as well. During wakefulness, hippocampal theta organizes place cell firing in sequences. This fine timing of hippocampal activity by theta oscillations is crucial for the encoding and subsequent consolidation of spatial memory through place cell replay during NREM sleep ripples (29). Comparatively, few studies have focused on how neuronal activity is structured during REM sleep, related or independently of theta oscillations (30–32). Transient increases of theta frequency and power during REM, referred to as phasic REM, are associated with an increase in firing rate and coordination throughout the hippocampus and with cortical areas (32, 33). Phasic REM has also been linked to the ponto-geniculo-occipital waves originating from the brainstem and suggested to coordinate various structures during REM sleep (34). To date, the link between these specific changes in REM sleep theta dynamics and behavior remains unclear. However, the coherence between theta oscillations in the hippocampus, medial prefrontal cortex (mPFC), and amygdala increases after aversive learning (35) in correlation with behavioral performance. The disruption of theta oscillations during REM sleep by optogenetically targeting the medial septum impairs hippocampus-dependent contextual memory consolidation (36). Additionally, the alteration of the activity of adult-born hippocampal neurons in the dentate gyrus specifically during REM sleep impaired contextual fear consolidation (37). While the manipulations did not affect theta oscillations, the fact that bidirectional modulation of firing impaired consolidation suggests the fine timing - potentially theta-paced-firing of newborn neurons is important. Moreover, slight structural modifications of synapses in newborn neurons were reported upon REM-sleep inhibition, indicative of weakened synapses. These results add to previous studies establishing that REM sleep promotes dendritic spine selective reinforcement or suppression in the neocortex (38). More work remains to be done to bridge the fine timing of patterned firing and theta oscillations during REM sleep with the observed structural plasticity in specific neuronal subpopulations and correlate it with behavioral outcomes.

## Sleep rhythms and plasticity: consolidation and homeostasis

Learning has been associated with Hebbian plasticity and synaptic potentiation (LTP). According to the synaptic homeostasis hypothesis (SHY), sleep plays a crucial role in homeostatic regulation by down-scaling synaptic weights to avoid saturation and allow for the formation of new memories during the subsequent wakefulness epoch. More specifically, this model predicts that global synaptic weights increase during wakefulness and decrease throughout sleep. While there is structural and molecular evidence for this process (39), it is difficult to assess structural changes and strength in synapses *in vivo* and in real time. Because cortical slow-wave activity stems from highly synchronized activity through UP and DOWN states, their amplitude is thought to reflect synaptic strength between cortical neurons. Accordingly, slow oscillations are strongest after extended wakefulness and progressively diminish across prolonged sleep episodes, in line with the SHY model (Fig. 3; (40)). Further, the changes in the slope of evoked potentials in the cortex, a marker of synaptic efficacy, are correlated with the changes in slow-wave activity, suggesting slow waves might contribute to synaptic downscaling (40). In parallel, the dynamics of firing rates across awake and sleep periods have been used as a proxy for neuronal excitability. Coherent with the SHY model, hippocampal cells, as a population, progressively increase their firing rate during waking (41, 42). During sleep, there is a global net decrease in firing rates but opposing trends between different stages: while overall spiking activity increases during NREM, it shows a marked decrease during REM (41, 42). Interestingly, the firing rate downregulation during REM could be predicted by spindles and SWR incidence during NREM (42). Finally, the canonical NREM sleep SWRs, for the longest time thought to be propitious to consolidation through LTP (43), also trigger LTD (44), and their inhibition prevents the normal decrease of evoked potentials across sleep, suggesting a potential role in homeostasis.

## Perspectives

Although simple to state, the link between sleep and memory actually translates into an incredibly complex field of research. First, sleep is not homogeneous and is subdivided into stages and substages characterized by different rhythms and patterns. Second, there are many different types of memories (episodic and semantic memories, procedural/skills memory, pavlovian conditioning, etc.) that rely on different, although sometimes overlapping, networks of structures, themselves exhibiting different sleep patterns. Further, episodic memories are not a complete and faithful representation of actual events. Episodic memory formation, therefore, encompasses the initial encoding of the information, modifications, merging with other memories, and even forgetting (45). Given the complexity of sleep, memory(ies), and the diversity of the involved structures, how do we design relevant basic research unraveling “the role of sleep for memory”?

In rodents, NREM sleep is traditionally studied as a homogeneous stage. Characterizing more specific NREM substages, or “microstates” potentially matching the 3 human NREM substages is an interesting avenue to link them with various aspects of memory processing up to the behavioral level. The function of phasic vs. tonic REM sleep in both humans and other species also remains to be investigated. In parallel, the study of patterns focuses on the function of specific network processes outside the frame of strictly defined stages. Indeed, several processes might co-exist within a stage and could be more reliably identified by linking them to specific patterns rather than the stage as a whole. The development of closed-loop systems and brain-machine interfaces for real-time pattern detection in neuronal firing, EEG or LFP signals brought about significant advancements in understanding the involvement of sleep patterns in memory formation (8–10, 13, 14, 17, 36). Sequences of place cells representing experienced trajectories are reactivated in subsequent sleep SWR (6) but to date, there is no causal evidence that the sequence *per se*, as opposed to the mere activation of the place-cell assembly (or engram) within a short time window, is important for memory consolidation. Testing theories on the significance of spike timing during patterns will require more precise real-time tools to perturb or impose the precise timing relationships between specific neurons without altering their firing rate at a broader time scale (46). In turn, clarifying the question of the relevance of the sequence itself would potentially reorient the field towards the nearly 80% of SWRs for which the associated neuronal content cannot be identified as statistically significant sequences by the current decoding algorithms. These could be reactivation events we are not yet capable of reading the way downstream reader brain structures do, or replay of remote memories not assessed by the experimenter. According to this hypothesis, the main function of SWR-related high synchronous events, including the ones we can’t decode, is to promote consolidation by means of memory replay. Another emerging and more integrated theory is that during sleep, the cortex and hippocampus enter default modes resulting from their physiological properties and hardwiring, involving bouts of heightened and synchronized activity (SWRs and UP states). These modes would primarily serve a homeostatic purpose (Fig. 3), but wakefulness activity and memory encoding would bias the precise timing of the firing during these events away from randomness, in which case specific memory traces could be consolidated (44, 47). Further, the bias induced by wakefulness activity would be stronger and more long lasting after learning or novelty (11) leading to periods of higher replay-to-noise ratio in SWR events. In that view, homeostasis and consolidation are on the same spectrum and heavily depend on the fine timing of the neuronal activity within the canonical sleep patterns.

Finally, reactivation, the main proposed mechanism for consolidation, is not universal in terms of structure and sleep stage, while homeostasis has been mostly studied in the neocortex. Therefore, more work needs to be done to precisely characterize sleep patterns in non-hippocampo-cortical structures that are involved in memory processing (i.e. amygdala, striatum). It is possible, and remains to be investigated, that consolidation and homeostatic processes differ or are absent in other structures, especially those with no detectable sleep reactivation and/or different firing rate distributions across brain states. This direction is especially interesting for the highly complex network of structures that have a controlling role over sleep states and transitions such as the pons, thalamus, hypothalamus, locus coeruleus, and basal forebrain. Indeed, in the same way that consolidation and homeostasis might be tightly related, control and function of the different sleep stages could also be linked (48).

Fueled by emerging recording, manipulation and analysis technologies with increasing spatio-temporal precision, we are in the process of completing a multidimensional knowledge space of mechanisms for different types of memory, different stages and substages of sleep and their associated physiological patterns. Although we might never reach a unifying theory for the memory function of sleep, expanding and precising this space will allow us to better integrate consolidation and homeostasis, unravel new links within memory function in all steps of memory formation from encoding to retrieval through consolidation, and link mnemonic mechanisms with other aspects of sleep such as sleep control, circadian rhythm or pathology.
